# The Effect of the Root Bark of *Lycium chinense* (Lycii Radicis Cortex) on Experimental Periodontitis and Alveolar Bone Loss in Sprague-Dawley Rats

**DOI:** 10.3390/antiox13111332

**Published:** 2024-10-31

**Authors:** Jinwon Yang, Hyosun Song, Jeongjun Lee, Hunsuk Chung, Young-Sam Kwon, Kyung-Hwan Jegal, Jae-Kwang Kim, Sae-Kwang Ku

**Affiliations:** 1 Department of Anatomy and Histology, College of Korean Medicine, Daegu Haany University, Gyeongsan 38610, Republic of Korea; yangjinwon@dhu.ac.kr; 2 Department of Veterinary Surgery, College of Veterinary Medicine, Kyungpook National University, Daegu 41566, Republic of Korea; legendx0070@knu.ac.kr (H.S.); kwon@knu.ac.kr (Y.-S.K.); 3 GAPI BIO Co., Ltd., Hwaseong 18622, Republic of Korea; orglab@gapibio.co.kr (J.L.); hunsukchung@dongbangchem.co.kr (H.C.); 4 Department of Korean Medical Classics, College of Korean Medicine, Daegu Haany University, Gyeongsan 38610, Republic of Korea; jegalkh@dhu.ac.kr; 5 Department of Physiology, College of Korean Medicine, Daegu Haany University, Gyeongsan 38610, Republic of Korea

**Keywords:** periodontitis, alveolar bone loss, anti-inflammatory, antioxidant, Lycii Radicis Cortex

## Abstract

Lycii Radicis Cortex (LRC), the dried root bark of *Lycium chinese* Mill., has traditionally been used as a medicinal herb in East Asia to treat fever and hyperhidrosis. In the present study, we investigated the effects of LRC extract on ligation-induced experimental periodontitis and associated alveolar bone loss in rats. Twenty-four hours after ligation placement, LRC was orally administered once daily for 10 days. Firstly, LRC administration inhibited anaerobic bacterial proliferation and inflammatory cell infiltration in gingival tissues. Additionally, LRC exhibited anti-inflammatory effects by reducing the expression of inflammatory mediators, including prostaglandin E_2_, interleukin-1β, and tumor necrosis factor-α. LRC treatment also downregulated mRNA expression of these inflammatory mediators in lipopolysaccharide-stimulated RAW 264.7 cells by inhibiting the mitogen-activated protein kinases and nuclear factor-κB (NF-κB) signaling pathways. Furthermore, LRC showed an antioxidant effect by decreasing the malondialdehyde level and inducible nitric oxide synthase activity in gingival tissues. Moreover, LRC effectively prevented the connective tissue degradation by inhibiting matrix metalloproteinase-8 expression and the loss of collagen-occupied areas in gingival tissues. LRC also decreased the receptor activator of NF-κB ligand/osteoprotegerin (RANKL/OPG) ratio, as well as the number and occupied areas of osteoclasts on the alveolar bone surface, thereby inhibiting alveolar bone loss. In summary, these findings suggest that LRC is a promising medicinal herb for alleviating periodontitis and related alveolar bone loss through its antimicrobial, anti-inflammatory, and antioxidant properties.

## 1. Introduction

Periodontitis is an inflammatory disease triggered by an imbalance in the oral microbiota and an abnormal host immune response, which ultimately leads to the destruction of the connective tissues and bone that support teeth [1]. With its high prevalence exceeding 10% among the global adult population, periodontitis presents a significant public health concern by diminishing quality of life through tooth loss and pain [2,3]. Particularly, the progression of attachment loss and tooth loss has been linked to socio-economic factors, such as low educational attainment and income levels, highlighting the necessity for developing simple and accessible preventive measures [4]. Moreover, epidemiological studies have highlighted the relationship between periodontitis and systemic diseases such as diabetes and cardiovascular diseases, in the terms of prevalence, progression, and severity [5,6]. As interest in the prevention and treatment of periodontitis continues to grow, research on natural products as adjuncts to overcome the limitations of conventional therapies such as antibiotics, scaling and root planning, is becoming increasingly active [7,8].

Inadequate oral hygiene facilitates the formation and persistence of bacterial biofilm, commonly known as dental plaque. Additionally, factors including individual susceptibility, host immune responses, and behavioral risks such as smoking disrupt the balance between resident microbes and the host’s immune system, triggering an inflammatory response in the gingival tissue [9]. The excessive release of proinflammatory mediators in gingival tissues leads to the infiltration of immune cells including polymorphonuclear neutrophils (PMNs), promoting their phagocytic activity and bacterial ingestion [10]. Gingivitis, the inflammation of gingiva, can further progress to periodontitis which is a chronic inflammatory condition that causes the destruction of connective tissue and alveolar bone. While the presence of neutrophils is essential for pathogen elimination, their hyperactivation can contribute to the destruction of periodontal connective tissue by releasing proteolytic enzymes such as matrix metalloproteinases (MMPs) [11]. Furthermore, the overproduction of cytokines, chemokines, and receptor activator of nuclear factor-kB ligand (RANKL) stimulates osteoclast formation, eventually contributing to alveolar bone loss [12].

Recently, oxidative stress has increasingly been recognized as a key factor in the development of periodontitis. When a pathogenic biofilm triggers an inflammatory response, neutrophils become the predominant immune cells in periodontal tissue and are the primary source of reactive oxygen species (ROS) in periodontitis [13]. Normally, ROS production by phagocytes is essential for the elimination of microbes. However, chronic and excessive production of ROS by neutrophils induces lipid peroxidation, generating metabolites such as malondialdehyde (MDA) and 4-hydroxynonenal [14]. This oxidative imbalance directly and indirectly promotes the persistence of inflammation and the destruction of periodontal tissue [15,16].

Lycii Radicis Cortex (LRC), the dried root bark of *Lycium chinense* Mill. or *L. barbarum* L. has long been used as a medicinal herb in East Asia to treat symptoms such as fever and hyperhidrosis. In traditional Chinese medicine (TCM), LRC has been employed to treat Yin-deficiency-heat syndrome, which results from heat generated internally due to Yin deficiency, often associated with prolonged illness and exhaustion [17]. According to TCM principles, periodontitis is categorized as a condition related to stomach-heat and kidney-yin deficiency syndromes [18,19,20], which is related to usages of LRC. In Korea, the “Kwangjebikeup”, a book published in 1790 and intended to provide remedies for rural communities with limited access to medical care, documents the use of a decoction of LRC for treating bleeding gums or gingivitis. Among the two source species of LRC, *L. chinense* Mill. is widely cultivated in various regions of Korea for use as functional food or medicinal herb [21]. Previous studies have reported the biological properties of LRC derived from *L. chinense* Mill. against muscle atrophy, glioblastomas, gastric ulcer [22,23,24]. In particular, numerous experimental studies have been conducted to evaluate its anti-osteoporosis effects on ovariectomy- and steroid-induced osteoporosis models [25,26,27]. However, the effect of LRC on periodontal diseases has not been investigated. Therefore, we investigated the protective effects of LRC (from *L. chinense* Mill.) on ligation-induced periodontitis in rats and explored the molecular mechanisms of its anti-inflammatory effect in lipopolysaccharide (LPS)-stimulated RAW 264.7 cells in the present study.

## 2. Materials and Methods

### 2.1. Preparation of LRC

The LRC extract powder was manufactured and supplied by Nutracore (Suwon, Republic of Korea) as described in the supplementary information (Batch No. LC-G221114). Some specimens of LRC extract (Code No. LRC2022Ku01) were deposited in the herbarium of the Medical Research center for Herbal Convergence on Liver Disease, Daegu Haany University (Gyeongsan, Republic of Korea). Kukoamine B was identified as a marker component using high-performance liquid chromatographic (HPLC) analysis (Supplementary Figure S1). Quantification of the peak area indicated that LRC contains 23 mg/g of kukoamine B.

### 2.2. Methods for In Vitro Experiments

#### 2.2.1. Cell Culture

RAW 264.7 cells were obtained from American Type Culture Collection (ATCC; Rockville, MD, USA). The cells were cultured in Dulbecco’s modified Eagle’s medium (DMEM; HyClone Laboratories, Logan, UT, USA) supplemented with 10% fetal bovine serum (Lonza, Walkersville, MD, USA) and 1% Antibiotic-Antimycotic solution (HyClone Laboratories), and were maintained in a CO_2_ incubator at 37°C with 5% CO_2_ under a humidified atmosphere.

#### 2.2.2. Measurement of Nitric Oxide (NO) Production

RAW 264.7 cells were pretreated with either LRC (0.3–3 mg/mL) or dexamethasone (1 μM, Sigma-Aldrich, St. Louis, MO, USA) for 1 h, followed by exposure to 0.3 μg/mL of LPS (Sigma-Aldrich) for 18 h. After the treatment period, 100 μL of conditioned media was mixed with an equal volume of Griess reagent (1% sulphanilamide, 0.1% *N*-(1-naphtyl)-ethylene diamine dihydrochloride, 5% phosphoric acid). The absorbance was measured at 540 nm using an automated microplate reader (EnSpire™, PerkinElmer, Waltham, MA, USA).

#### 2.2.3. Measurement of Prostaglandin E_2_ (PGE_2_) Production

PGE_2_ levels in the conditioned media were measured using a commercial competitive enzyme-linked immunosorbent assay (ELISA) kit (Prostaglandin E2 Parameter Assay Kit, R&D Systems, Minneapolis, MN, USA). Briefly, conditioned media and primary PGE_2_ antibody were added to a goat anti-mouse-coated 96-well plate. After incubation on a microplate shaker, horseradish peroxidase (HRP)-labeled PGE_2_ was added, and color development was initiated using a substrate solution. The reaction was stopped by adding 2 N H_2_SO_4_ solution. Absorbance was measured at a wavelength of 450 nm using an automated microplate reader (EnSpire™).

#### 2.2.4. RNA Isolation and RT-qPCR

Total RNA was isolated using a TRI-Solution^TM^ (Bioscience Technology, Daegu, Republic of Korea). An amount of 2 μg of RNA was reverse transcribed using oligo (dT) primer, Accupower^®^ RT PreMix (Bioneer, Daejeon, Republic of Korea), and a SimpliAmp^TM^ Thermal Cycler (Applied Biosystems, Waltham, MA, USA). Synthesized cDNA was amplified using SyBr green Ex-Taq master mix (Takara, Shiga, Japan), Quantstudio 5 Thermal cycler (Applied Biosystems), and specific primers for inducible *nitric oxide synthase* (*iNOS*), *cyclooxygenase-2* (*COX-2*), *tumor necrosis factor-α* (*TNF-α*), *interleukin* (*IL*)-*1β*, *monocyte chemoattractant protein-1* (*MCP-1*), and *glyceraldehyde-3-phosphate dehydrogenase* (*GAPDH*). All sequences of the oligonucleotide primers used for PCR are listed in Table 1. After PCR amplification, a melting curve of each amplicon was determined to verify its accuracy. The expressions of each gene were normalized by *GAPDH* mRNA expression, using the comparative threshold cycle method [28].

#### 2.2.5. Immunoblot Analysis

Cells were lysed with radioimmunoprecipitation assay buffer (RIPA) containing sodium fluoride, β-glycerophosphate, sodium orthovanadate, sodium pyrophosphate, and a protease inhibitor cocktail (GenDEPOT, Barker, TX, USA) and then incubated on ice for 1 h. After centrifugation at 15,000× *g* for 10 min, the supernatant was collected as whole cell lysates. Protein concentrations were determined using a bicinchoninic acid (BCA) assay (Thermo Fischer Scientific, Waltham, MA, USA). Equal amounts of proteins were resolved by sodium dodecyl sulfate-polyacrylamide gel electrophoresis (SDS-PAGE) and transferred to a nitrocellulose membrane (GE Healthcare Life Sciences, Buckinghamshire, UK). After blocking with 5% bovine serum albumin, the membrane was sequentially reacted with primary and secondary antibodies. Immunoreactive proteins were detected using West-Q Pico ECL solution (GenDEPOT) and visualized with a Fusion FX7 (Vilber Lourmat, Marne-la-Vallée, France). Densitometric analysis was performed using Image J software (ver. 1.53). Band intensity values were calculated by dividing the intensity of phosphorylated protein by the intensity of total target protein. Primary antibodies against phosphorylated c-Jun N-terminal kinase (JNK) 1/2, JNK 1/2, phosphorylated p38, p38, phosphorylated extracellular-signal regulated protein kinase (ERK) 1/2, ERK 1/2, phosphorylated p65, as well as HRP-conjugated secondary antibodies were obtained from Cell Signaling Technology (Danvers, MA, USA). The anti-p65 antibody was purchased from Santa Cruz Biotechnology (Santa Cruz, CA, USA).

#### 2.2.6. Measurement of Radical Scavenging Activity

Radical scavenging activity was assessed using 2,2-diphenyl-1-picrylhydrazyl (DPPH). LRC was dissolved in water with concentrations ranging from 10 to 300 μg/mL. A 20 μL aliquot of each LRC sample was mixed with 180 μL of DPPH solution (150 μM, dissolved in ethanol) and incubated at room temperature for 30 min, protected from light. Absorbance was measured at a wavelength of 517 nm using an automated microplate reader (EnSpire™). The radical scavenging activity was calculated by comparing the absorbance of the DPPH solution with (S) and without the samples (S_0_) to the absorbance of the solvent with (C) and without the sample (C_0_). The percentage of radical scavenging activity is determined using the following equation: [(S − S_0_)/(C − C_0_)] × 100 (%).

### 2.3. Methods for In Vivo Experiments

#### 2.3.1. Preparation of Test Materials

The LRC powder was first dissolved in distilled water at a concentration of 40 mg/mL and further diluted in distilled water to concentrations of 20 and 10 mg/mL for the administration. Additionally, indomethacin (IND; Sigma-Aldrich) was prepared by suspending it in distilled water at a concentration of 1 mg/mL. Insadol^TM^ Plus (INP; Dongkook Pharmaceutical, Seoul, Republic of Korea), commonly used in the Korean market as a pharmaceutics agent for the relief of periodontal disease, was prepared by grinding tablets and suspending in distilled water at a concentration of 12.6 mg/mL.

#### 2.3.2. Animal Husbandry and Grouping

A total of 70 six-week-old male Sprague-Dawley (Crl:CD) rats (body weight ranged from 150 to 210 g upon receipt) were obtained from OrientBio (Seungnam, Republic of Korea) and acclimated for 9 days. Animals were then divided into 7 groups (10 rats/group): intact vehicle control group, experimental periodontitis (EPD) control group, IND (EPD with IND oral administration) group, INP (EPD with INP oral administration) group, and three LRC groups (EPD with LRC administration at doses of 50, 100, and 200 mg/kg, respectively). All animal experiments were conducted according to the national regulation of the usage and welfare of laboratory animals and approved by the Institutional Animal Care and Use Committee in Daegu Haany University (Approval No. DHU2022-101).

#### 2.3.3. Induction of EPD and Administration of Test Materials

EPD was induced by placing a sterile nylon suture (3–0) around the cervix of the upper left incisor in rats. The suture was tied on the buccal side of the tooth, based on the established method [29,30]. In the intact vehicle control groups, we only examined the cervix of the upper left incisor tooth, without applying any ligation. The procedures were conducted under inhalation anesthesia with 2–3% isoflurane (Hana Pharm. Co., Hwaseong, Republic of Korea) using a rodent inhalation anesthesia apparatus (Surgivet, Waukesha, WI, USA) and a rodent ventilator (Model 687, Harvard Apparatus, Cambridge, UK).

A total of 24 h after ligation placement, test materials including IND, INP, or three doses of LRC were administered orally by gastric gavage using a stainless steel zonde at a volume of 5 mL/kg, once daily for 10 consecutive days. To provide the same restraint stress for the intact vehicle control group and the EPD control group, an equal volume of distilled water was administered instead of IND, INP, or LRC.

#### 2.3.4. Measurements of Alveolar Bone Loss Scores

The maxillary bone including the area with the ligation placement was photographed with a digital camera, and then excised. The distance from the cusp tip to the alveolar bone, indicative of horizontal alveolar bone loss, was measured using an electronic digital caliper along the axis of the root of the upper left incisor tooth as in previously described methods [29,31] and recorded in millimeters per rat.

#### 2.3.5. Microbiological Analysis

The buccal gingival tissues from the ligated area were carefully excised and immediately homogenized with 0.3 mL of Brain Heart Infusion (BHI) broth. The homogenized tissue samples were then diluted 1:100 and 1:1000, and plated on BHI agar supplemented with defibrinated sheep blood to culture anaerobic bacteria. The plates were incubated at 37 °C for using anaerojars and anaerogen sachets. After 48 h, the numbers of the formed colonies were counted and represented as ×10^2^ CFU/g tissues [32].

#### 2.3.6. Measurement of Myeloperoxidase (MPO) Activity

MPO activity was measured using a spectrophotometric assay [33]. The buccal gingival tissues around the left incisor were excised and suspended in 0.5% hexadecyltrimethyl-ammonium bromide (Gibco, Carlsbad, CA, USA) in 50 mM potassium phosphate buffer (pH 6.0) to solubilize MPO, and then homogenized. After two freeze–thaw cycles, the homogenate was incubated with additional buffer (400 μL/15 mg of tissue) for 12 min, then centrifuged at 1000× *g* for 12 min at 4 °C. The supernatant (0.1 mL) was mixed with 2 mL of phosphate buffer (50 mM, pH 6.0), containing 0.167 mg/mL *o*-dianisidine dihydrochloride (Sigma-Aldrich), distilled water, and 0.0005% hydrogen peroxide. Absorbance was measured at 460 nm using a UV/Vis spectrometer (OPTIZEN POP, Mecasys, Daejeon, Republic of Korea). One unit of activity was defined as the amount needed to degrade 1 μM of hydrogen peroxide per min at 25 °C, and the results were expressed as units per mg of tissue.

#### 2.3.7. Measurement of PGE_2_, MMP-8, TNF-α, and IL-1β

To measure buccal gingival expressions of PGE_2_, MMP-8, TNF-α, and IL-1β, tissues around the ligation placement were collected and homogenized. Each concentration of PGE_2_ (R&D Systems), MMP-8 (MyBioSource, San Diego, CA, USA), TNF-α, and IL-1β (Abcam, Cambridge, UK) in the tissue homogenates was measured using a commercial ELISA kit according to the manufacturer’s instructions.

#### 2.3.8. RT-qPCR Analysis for *RANKL* and *Osteoprotegerin* (*OPG*) mRNA Expressions

The *RANKL* and *OPG* mRNA expressions in the maxillary gingival tissues were detected using RT-qPCR. Total RNA was extracted using Trizol reagent^®^ (Invitrogen, Carlsbad, CA, USA). The extracted RNA was reverse transcribed using the High-Capacity cDNA Reverse Transcription Kit (Thermo Fisher Scientific). And the synthesized cDNA was amplified using the CFX96^TM^ Real-Time System (Bio-Rad, Hercules, CA, USA). All sequences of the oligonucleotide primers used for PCR are listed in Table 1. The expressions of each gene were normalized by *β-actin* mRNA expression, using the comparative threshold cycle method [28].

#### 2.3.9. Measurement of Lipid Peroxidation

Lipid peroxidation in the buccal gingival tissue was assessed by measuring the MDA levels. Buccal gingival tissues from the ligature placement site were collected and homogenized in a buffer composed of 50 mM Tris-HCl, 0.1 mM EGTA, and 1 mM phenylmethylsulfonylfluoride (pH 7.4). A 100 μL aliquot of the tissue homogenate was then added to a reaction mixture containing SDS, acetic acid, thiobarbituric acid, and distilled water. The samples were incubated at 95 °C for 1 h, and subsequently centrifuged at 3000× *g* for 10 min at 4 °C. The absorbance of the supernatant was measured at 650 nm by a UV/Vis spectrometer. Results are expressed as μM per mg of tissue.

#### 2.3.10. Measurement of Inducible iNOS Activity

The gingival activity of iNOS was assessed by measuring the conversion of L-[^3^H]-arginine to L-[^3^H]-citrulline in tissue homogenates, following established protocols [29,30,34]. Briefly, tissue homogenates were incubated with a reaction mixture containing L-[^3^H]-arginine (10 mM, 5 kBq/tube), NADPH (1 mM), calmodulin (30 nM), tetrahydrobiopterin (5 mM), and calcium (2 mM) for 30 min at 22 °C. The reaction was terminated by the addition of 0.5 mL of ice-cold HEPES buffer (pH 5.5) containing EGTA (2 mM) and EDTA (2 mM), which act as calcium chelators. To determine NOS-independent activity, the formation of L-[^3^H]-citrulline was measured in the absence of NADPH. Additionally, to evaluate calcium-independent NOS activity, reactions were performed with NADH (1 mM) in the absence of calcium and in the presence of EGTA (5 mM). The reaction mixtures were then passed through Dowex 50W (Na^+^ form) columns to separate L-[^3^H]-citrulline, which was quantified using a liquid scintillation counter (Wallac, Annapolis, MD, USA). iNOS activity is expressed as fM/mg/min.

#### 2.3.11. Histopathological Analysis

After the sacrifice, the maxilla region around the placed ligature including the left and right incisors was excised. After fixation in 10% neutral buffered formalin, tissues were incubated in a decalcifying solution containing 24.4% formic acid and 0.5 N sodium hydroxide for 5 days. Decalcified tissues were cross trimmed, embedded in paraffin, and sectioned at a thickness of 3–4 μm. Representative sections were then stained with hematoxylin and eosin for histological observation. The regions between the left and right incisor teeth, where the ligature was applied, were evaluated histologically using a previously established scoring system (0 to 3) [29,30,35], considering the infiltration of inflammatory cells and the integrity of the alveolar bone and cementum. In addition, the numbers of infiltrated inflammatory cells (numbers/mm^2^) and collagen-occupied areas (%/mm^2^) on the gingival tissues around the left incisor teeth were quantified. Furthermore, the volume of alveolar bone (%/mm^2^), as well as the numbers (numbers/mm^2^) and occupied percentages of osteoclasts (%/mm^2^) on the alveolar bone surface between the upper incisor teeth were also measured. Histological analyses were conducted using a light microscope (Eclipse 80*i*, Nikon, Tokyo, Japan)-equipped histological camera system (ProgRes^TM^ C5, Jenoptik Optical Systems GmbH, Jena, Germany) and computer-assisted image analyzer (*i*Solution FL ver 9.1, IMT *i*-solution Inc., Bernaby, BC, Canada), with the histopathologist remaining blinded to the group assignments during evaluation.

### 2.4. Statistical Analysis

All numeric data were expressed as mean ± standard deviation. A one-way analysis of variance (ANOVA) test was performed to determine the statistical significance of differences among the experimental groups. According to the variance homogeneity result by Levene’s test, Tukey’s honestly significant difference (HSD) or Dunnett’s T3 test were conducted as a *post hoc* analysis. *p* values under 0.05 were considered statistically significant. All statistical analyses were performed using SPSS 18.0 software (SPSS, Chicago, IL, USA).

## 3. Results

### 3.1. Anti-Inflammatory Effect of LRC on the LPS-Stimulated RAW 264.7 Cells

#### 3.1.1. LRC Decreased the Expression of Proinflammatory Mediators

**Figure 1:**
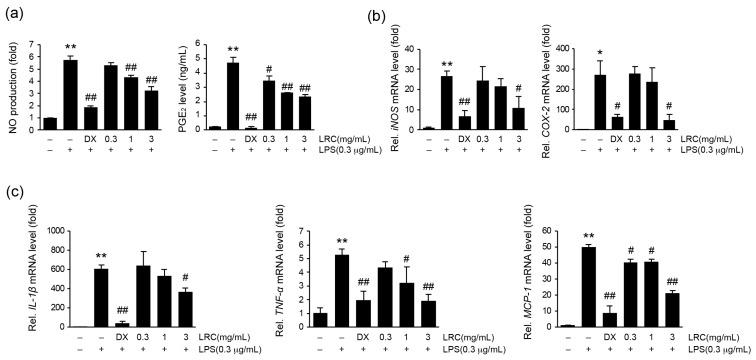
Effect of Lycii Radicis Cortex extract (LRC) on inflammatory mediators’ expression in lipopolysaccharide (LPS)-stimulated RAW 264.7 cells. RAW 264.7 cells were pretreated with LRC (0.3–3 mg/mL) or dexamethasone (DX, 1 μM) for 1 h, and further stimulated with lipopolysaccharide (LPS, 0.3 μg/mL) for (**a**) 18 h or (**b**,**c**) 6 h. After treatment, (**a**) levels of nitric oxide (NO) and prostaglandin E_2_ (PGE_2_) were detected in collected conditioned media. (**b**) Relative mRNA levels of *iNOS*, *COX-2*, (**c**) *IL-1β*, *TNF-α*, and *MCP-1* were measured using RT-qPCR. The expressions of each gene were normalized by the expression of *GAPDH*. Significant versus vehicle group, * *p* < 0.05, ** *p* < 0.01; versus LPS-treated group, ^#^ *p* < 0.05, ^##^ *p* < 0.01.

To confirm the anti-inflammatory effect of LRC, we conducted in vitro experiments using LPS-stimulated RAW 264.7 cells. Prior to evaluating the anti-inflammatory effect of LRC, we assessed the cytotoxicity of LRC on HaCaT (a human keratinocyte cell), HDFn (a human primary dermal fibroblast-neonatal cell), and RAW 264.7 (a murine macrophage-derived cell) cells. As a result, no significant changes on the survivability of HaCaT, HDFn, and RAW 264.7 cells by LRC treatment were observed (Supplementary Figure S2). To investigate the inhibitory effect of LRC on NO and PGE_2_ production, RAW 264.7 cells were pretreated with LRC (0.3–3 mg/mL) or dexamethasone (1 μM, as a positive control) for 1 h. And cells were further stimulated with LPS (0.3 μg/mL, 18 h). After treatment, the levels of NO and PGE_2_ in the conditioned media were measured. As expected, LPS stimulation significantly increased the production of NO and PGE_2_ compared to the vehicle-treated group. However, LRC (1 and 3 mg/mL) pretreatment significantly reduced the NO and PGE_2_ production (Figure 1a). Similarly, the LPS-induced increased mRNA levels of *iNOS* and *COX-2*, enzymes responsible for producing NO and PGE_2_, respectively, were successfully decreased by LRC (3 mg/mL) treatment (Figure 1b). IL-1β, TNF-α, and MCP-1, produced from innate immune cells including macrophages, have been known as major inflammatory mediators for exaggerating acute inflammation [36]. To investigate the effects of LRC on the production of proinflammatory cytokines by LPS, RAW 264.7 cells were pretreated with LRC (0.3–3 mg/mL) for 1 h, and subsequently exposed to LPS (0.3 μg/mL, 6 h). As a result, the increased mRNA levels of proinflammatory cytokines including *IL-1β*, *TNF-α*, and *MCP-1* by LPS stimulation were dose-dependently reduced by LRC treatment (Figure 1c).

#### 3.1.2. LRC Inhibited the Activation of Mitogen-Activated Protein Kinases (MAPKs) and Nuclear Factor-κB (NF-κB) Signaling Pathways

**Figure 2:**
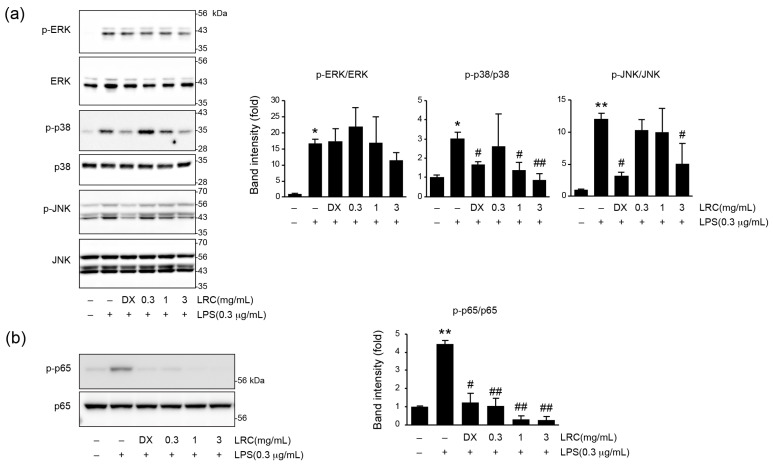
Effect of LRC on mitogen-activated protein kinases (MAPKs) and nuclear factor-κB (NF-κB) activation in LPS-stimulated RAW 264.7 cells. For immunoblot analysis, RAW 264.7 cells were pretreated with LRC (0.3–3 mg/mL) for 1 h and further incubated with LPS (0.3 μg/mL, 0.5 h). (**a**) MAPKs phosphorylation. (**b**) p65 phosphorylation. Significant versus vehicle group, * *p* < 0.05, ** *p* < 0.01; versus LPS-treated group, ^#^ *p* < 0.05, ^##^ *p* < 0.01.

To elucidate the molecular mechanism underlying the anti-inflammatory effects of LRC, we investigated its effect on MAPK and NF-κB signaling pathways (Figure 2). For the immunoblotting, cells were pretreated with LRC (0.3–3 mg/mL) for 1 h, and further incubated with LPS (0.3 μg/mL, 0.5 h). In the MAPK signaling pathway, LRC treatment at 1 and 3 mg/mL significantly reduced the phosphorylation of p38. And the phosphorylation of JNK was significantly reduced by only 3 mg/mL of LRC treatment. But no significant effect of LRC (0.3–3 mg/mL) treatment on the phosphorylation of ERK was observed (Figure 2a). In the NF-κB pathway, the increase in p65 phosphorylation by LPS, which indicates transcriptional activation of NF-κB [37], was significantly and dose-dependently reduced by LRC (0.3–3 mg/mL) treatment (Figure 2b). These results suggest that LRC exhibits anti-inflammatory effects by inhibiting the production of inflammatory mediators through the suppression of MAPK and NF-κB signaling pathways.

### 3.2. The Effect of LRC on the EPD Ligation Rats

#### 3.2.1. Experiment Procedure and Body Weight Gains

**Figure 3:**
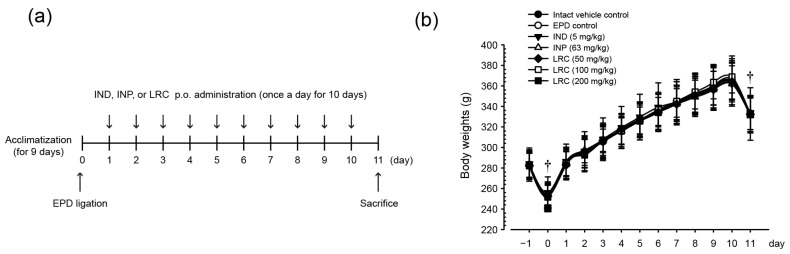
Procedure of experiment and body weights. (**a**) Scheme of animal experiment. At 24 h after experimental periodontitis (EPD) ligation, rats (10/group) were orally administered with indomethacin (IND, 5 mg/kg), Insadol Plus^TM^ (INP, 63 mg/kg), or three doses of LRC (50, 100, or 200 mg/kg) once a day for 10 days. Day 0 means the day of ligature placement. (**b**) Animal body weights. Body weight was measured from day −1 to day 11. All values are expressed as mean ± SD of ten rats. ^†^ All rats were fasted overnight before both the ligature placement and sacrifice.

EPD was induced on day 0 by placing a sterilized nylon thread ligature around the upper left incisor of rats. At 24 h after the ligation placement, IND (5 mg/kg), INP (63 mg/kg), and three different doses of LRC (50, 100, and 200 mg/kg) were administered orally once a day for 10 consecutive days. On day 11, all animals were sacrificed, and tissues were harvested (Figure 3a). Over the 11 days of the experimental period, there were no significant body weight changes in the EPD control group compared to the intact vehicle control group in the present study. Similarly, rats administered with IND, INP, or LRC (50, 100, and 200 mg/kg) did not show any significant changes in body weight compared to the EPD group (Figure 3b).

#### 3.2.2. LRC Ameliorated Alveolar Bone Loss Scores

**Figure 4:**
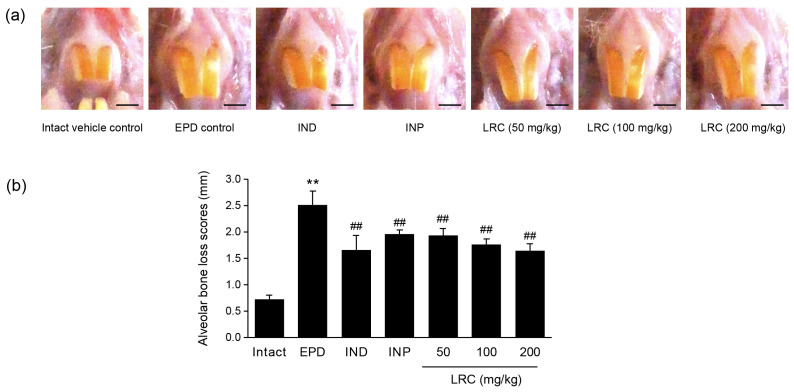
Effect of LRC on the alveolar bone loss. (**a**) Representative photos taken from upper left incisor teeth. Scale bars indicate 3.00 mm. (**b**) Alveolar bone loss scores. Horizontal alveolar bone loss, the distance from the cusp tip to the alveolar bone, was measured. All values are expressed as mean ± SD of ten rats. Significant versus intact vehicle control group, ** *p* < 0.01; versus EPD control group, ^##^ *p* < 0.01.

Exposed teeth root areas were detected as the alveolar bone loss scores. In the EPD control group, alveolar bone loss scores were significantly increased compared to the intact vehicle control. As positive controls, both the IND (5 mg/kg) and the INP (63 mg/kg) groups exhibited a significant reduction in alveolar bone loss scores compared to the EPD group. All three dosages of LRC (50, 100, and 200 mg/kg) also showed a significant reduction in alveolar bone loss scores against ligation-induced EPD. In particular, the 200 mg/kg of LRC treatment showed a favorable inhibitory effect on ligation-induced EPD, comparable to those of IND or INP administration (Figure 4).

#### 3.2.3. LRC Decreased Gingival Anaerobic Bacterial Count and MPO Activity

**Figure 5:**
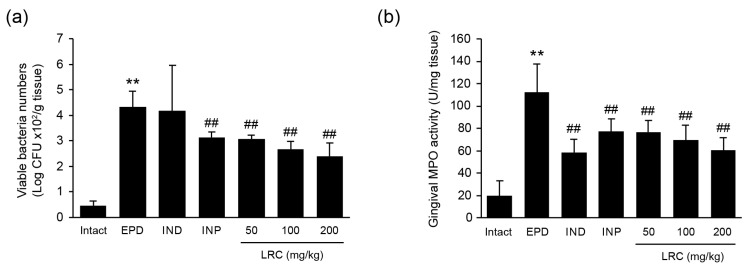
Effect of LRC on the buccal gingival total anaerobic bacterial counts and myeloperoxidase (MPO) activity. (**a**) Viable bacteria numbers. Excised buccal gingival tissues were homogenized and diluted for plating BHI agar to culture anaerobes. Formed colonies were counted as ×10^2^ CFU/g tissue. (**b**) MPO activity in gingival tissues. One unit of activity was defined as the amount required to degrade 1 μM of hydrogen peroxide per min at 25°C, with results expressed in units per milligram of tissue. All values are expressed as mean ± SD of ten rats. Significant versus intact vehicle control group, ** *p* < 0.01; versus EPD control group, ^##^ *p* < 0.01.

In the EPD control group, the number of total anaerobic bacteria in the buccal gingiva was significantly higher compared to the intact vehicle control group. Administration of all three doses of LRC (50, 100, and 200 mg/kg) significantly reduced the viable bacterial count. However, IND (5 mg/kg) showed no significant changes in anaerobic bacterial count against ligation-induced EPD. Notably, LRC at 100 and 200 mg/kg showed a more favorable inhibitory effect on the total anaerobic bacteria in the buccal gingiva, comparable to that of INP (63 mg/kg) in the current experiment (Figure 5a). To measure neutrophil accumulation in gingival tissues, the MPO activity in tissue homogenates was assessed. Gingival MPO activity was significantly increased in the EPD control group compared to the intact vehicle control group. However, administration with all three doses of LRC (50, 100, and 200 mg/kg) significantly and dose-dependently decreased MPO activity. Specifically, LRC at 200 mg/kg showed the most potent inhibitory effect on ligation-induced EPD, comparable to that observed in the IND group (Figure 5b).

#### 3.2.4. LRC Decreased the Gingival Expression of Proinflammatory Mediators

**Figure 6:**
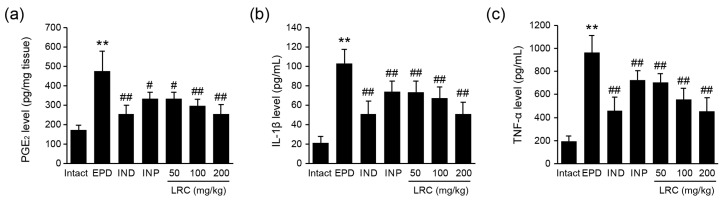
Effect of LRC on the gingival expression of inflammatory mediators. The expression levels of (**a**) PGE_2_, (**b**) IL-1β, and (**c**) TNF-α in the gingival tissues were measured using a commercial ELISA kit. All values are expressed as mean ± SD of ten rats. Significant versus intact vehicle control group, ** *p* < 0.01; versus EPD control group, ^#^ *p* < 0.05, ^##^ *p* < 0.01.

The buccal gingival expressions of proinflammatory mediators including PGE_2_, IL-1β, and TNF-α were also detected using ELISA. Consistent with the findings from in vitro experiments, increased levels of PGE_2_, IL-1β, and TNF-α in the EPD control group were significantly reduced by administration of all three doses of LRC (50, 100, and 200 mg/kg). In particular, LRC administration at 200 mg/kg showed favorable inhibitory effects on the gingival expression of PGE_2_, IL-1β, and TNF-α as comparable to those of IND (5 mg/mL) group (Figure 6).

#### 3.2.5. LRC Decreased MDA Levels and iNOS Activity

**Figure 7:**
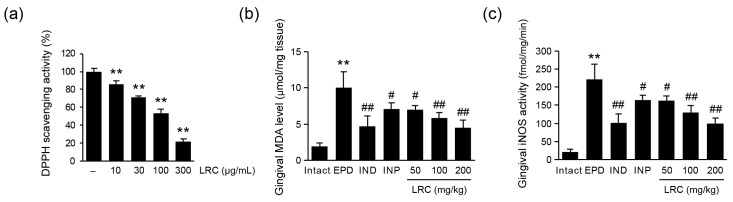
Effect of LRC on oxidative stress. (**a**) DPPH (2,2-diphenyl-1-picrylhydrazyl) radical scavenging activity. Significant versus vehicle group, ** *p* < 0.01. (**b**) Malondialdehyde (MDA) level and (**c**) iNOS activity were assessed in gingival tissue. All values are expressed as mean ± SD of ten rats. Significant versus intact vehicle control group, ** *p* < 0.01; versus EPD control group, ^#^ *p* < 0.05, ^##^ *p* < 0.01.

Oxidative stress is considered one of the key mechanisms involved in the progression of periodontal disease [16]. To investigate the antioxidant properties of LRC, we first evaluated its radical scavenging activity using the DPPH assay (Figure 7a). LRC exhibited a dose-dependent increase in radical scavenging activity, with statistically significant differences observed at concentrations ranging from 10 to 300 μg/mL. Additionally, we measured the MDA level in the gingival tissues as a maker of lipid peroxidation (Figure 7b). The ligation placement led to a significant increase in MDA level in the gingival tissue of the EPD control group compared to the intact vehicle control group. However, the administration of LRC (50, 100, and 200 mg/kg) significantly and dose-dependently reduced gingival MDA levels. Notably, LRC at 200 mg/kg showed the most potent inhibitory effect on ligation-induced MDA levels, comparable to that observed in the IND (5 mg/kg) group. Moreover, we assessed the gingival activity of iNOS, which is primarily responsible for NO formation in periodontitis (Figure 7c). NO not only indicates the intensity of the inflammatory response within tissues, but also reflects the level of oxidative stress. Excessive NO production by phagocytic cells can lead to the formation of NO derivatives such as peroxynitrite, contributing to tissue damage through oxidation and nitrosylation [38]. In the present experiments, ligation significantly increased gingival iNOS activity in the EPD control group compared to the intact vehicle group. Similarly to NO production and *iNOS* mRNA levels in LPS-stimulated RAW 264.7 cells (Figure 1), administration of LRC (50, 100, and 200 mg/kg) significantly reduced elevated iNOS activity. Specifically, LRC at 200 mg/kg exhibited the most potent inhibitory effect, comparable to that observed in the IND group.

#### 3.2.6. LRC Decreased MMP-8 Levels in Gingival Tissue

**Figure 8:**
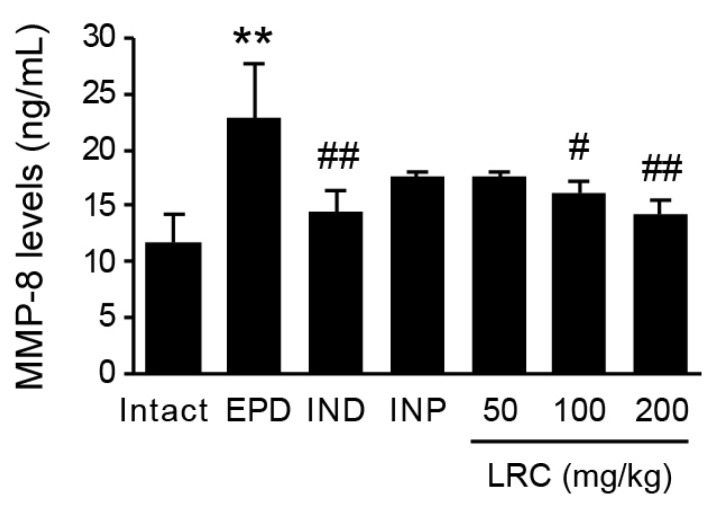
Effect of LRC on matrix metalloproteinase-8 (MMP-8) expression. MMP-8 level in gingival tissues was assessed using a commercial ELISA kit. All values are expressed as mean ± SD of ten rats. Significant versus intact vehicle control group, ** *p* < 0.01; versus EPD control group, ^#^ *p* < 0.05, ^##^ *p* < 0.01.

MMPs, proteolytic enzymes, are responsible for the degradation of periodontal connective tissue. Since type I collagen is a major component of periodontal extracellular matrix, the role of MMP-8 in periodontal tissue destruction has received significant attention. Previous studies have shown that the concentration of MMP-8 is associated with the severity of tissue destruction, suggesting its potential as a biomarker for periodontal disease [11]. Therefore, we assessed the MMP-8 level in gingival tissue using ELISA (Figure 8). The ligation placement significantly increased the MMP-8 level in the gingival tissue of the EPD control group compared to the intact vehicle control group. However, the administration of LRC dose-dependently reduced gingival MMP-8 levels with statistically significant differences observed at 100 and 200 mg/mL. Notably, LRC at 200 mg/kg showed the most potent inhibitory effect on ligation-induced MMP-8 level, comparable to that observed in the IND (5 mg/kg) group.

#### 3.2.7. LRC Decreased RANKL/OPG Ratio

**Figure 9:**
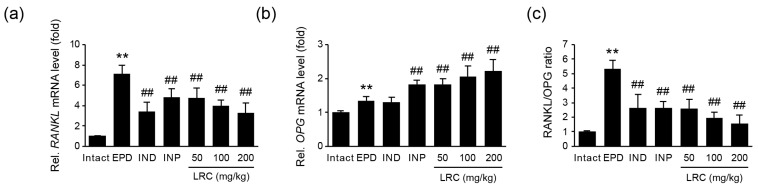
Effect of LRC on *receptor activator of nuclear factor-κB ligand* (*RANKL*) and *osteoprotegerin* (*OPG*) mRNA expressions. Relative mRNA levels of (**a**) *RANKL* and (**b**) *OPG* in gingival tissues were measured using RT-qPCR. The expressions of each gene were normalized by the expression of *β-actin*. All values are expressed as mean ± SD of ten rats. (**c**) RANKL/OPG ratio. The mRNA level of *RANKL* was divided by that of *OPG*. Significant versus intact vehicle control group, ** *p* < 0.01; versus EPD control group, ^##^ *p* < 0.01.

The RANKL/RANK/OPG system plays a crucial role in the maturation of osteoclasts, as well as in bone remodeling. Therefore, we assessed the mRNA expression of *RANKL* and *OPG* in gingival tissue (Figure 9). Ligation placement significantly increased the *RANKL* mRNA level in the gingival tissue of the EPD control group compared to the intact vehicle control group. However, the administration of LRC (50, 100, and 200 mg/kg) significantly and dose-dependently reduced gingival *RANKL* mRNA levels (Figure 9a). In contrast, the administration of LRC (50, 100, and 200 mg/kg) significantly increased the mRNA level of *OPG* compared to the EPD group (Figure 9b). Next, since an increase in the RANKL/OPG ratio is known to reflect the occurrence of periodontitis [39], we represented the RANKL/OPG ratio by normalizing the *RANKL* mRNA level to the *OPG* mRNA level (Figure 9c). The ratio, which increased due to ligation, was significantly and dose-dependently decreased with LRC administration (50, 100, and 200 mg/kg).

#### 3.2.8. Histopathological Changes of Maxillary Regions

**Figure 10:**
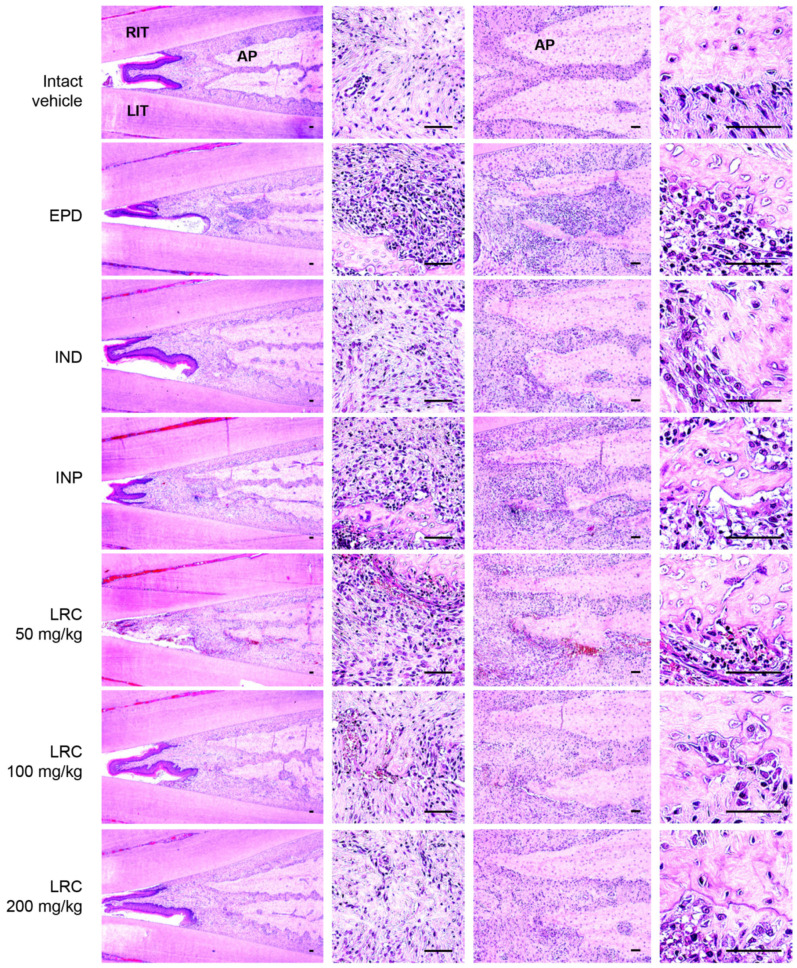
Representative histological images of gingival tissue and alveolar bone areas. Tissues were stained with hematoxylin and eosin. Images of gingival tissue and alveolar bone areas were captured between upper incisor teeth. Scale bars indicate 50 μm. RIT, right incisor tooth; LIT, left incisor tooth; AP, alveolar process.

Histopathological analyses were performed on both the gingival tissue and alveolar bone regions (Figure 10). The results showed a significant increase in inflammatory cell infiltrations, mostly PMNs, within the gingival tissues located between the upper left and right incisors in the EPD group. This was accompanied by severe edematous changes, including the loosening of collagen fibers and a loss of their compactness. In the alveolar bone regions of the EPD group, there was notable activation of osteoclasts, characterized by an increase in the numbers of osteoclast cells and the percentage of the alveolar bone surface occupied by these cells (OC/BS), along with a marked decrease in alveolar processes. These findings suggest that periodontitis and related bone loss were induced by the ligature placement, as previously reported [29,30].

However, oral administration of LRC at all doses (50, 100, and 200 mg/kg) significantly and dose-dependently reduced ligation-induced histopathological changes in gingival tissues, including histological scores, inflammatory cell infiltrations, and regions occupied by collagen fibers (Table 2). Moreover, all doses of LRC significantly restored alveolar bone volumes, and reduced the numbers and percentages of osteoclast-occupied regions on the alveolar bone surface (Table 3). Notably, LRC at 200 mg/kg exhibited a strong inhibitory effect on ligation-induced histological changes in the gingival tissue and alveolar bone regions, comparable to the effects observed with IND (5 mg/kg).

## 4. Discussion

Along with conventional approaches, herbal medicine and natural phytochemicals have gained attention for their efficacy, safety, and cost effectiveness as adjuncts [7]. Despite the ethnopharmacological use of LRC, research validating its efficacy against periodontitis and related alveolar bone loss through animal studies remains limited. Therefore, we investigated the preventive and therapeutic efficacy of LRC on ligation-induced EPD rats. While molars are often preferred for EPD induction due to their clinical relevance including severe inflammation [40], we selected an incisor ligation model to align with our focus on developing preventive measures using medicinal herbs, aiming for a milder model. The incisor ligation model offers improved visibility and ease of procedure, and histopathological and biochemical analyses have confirmed its ability to induce periodontal inflammation and alveolar bone resorption [40,41]. Additionally, the incisor ligation model has been effectively used in prior studies on natural products and herbs, including our own previous research, to evaluate their efficacy [29,30,42,43]. In the present study, INP (containing Magnolia bark 75% ethanol soft extract) was used as a positive control representing a natural preventive agent, while IND (a non-selective cyclooxygenase inhibitor) served as a positive control representing anti-inflammatory agents.

Periodontal disease is initiated by localized gingival inflammation, which arises from the surge of pathogenic bacteria in the dental plaque and the subsequent host responses [10]. Because the outgrowth of keystone pathogens within the oral biofilm contributes to the pathogenesis of periodontitis, antimicrobial approaches using antibiotics are conventionally employed with scaling and root planning [44]. However, the systemic and direct use of antibiotics presents challenges such as disruption of the gut microbiota and development of antibiotic resistance [45]. Therefore, as one of the alternative options, natural products with antimicrobial effects have been investigated as potential candidates [8]. In our experiment, ligature placement significantly increased the anaerobic bacteria count as previously reported [29,30]. However, LRC administration showed a significant inhibitory effect on the viable anaerobic bacteria number (Figure 5a). Although antimicrobial effects have been reported for some isolated ingredients of LRC [46], further research on LRC and its constituents targeting specific keystone anaerobic bacteria related to periodontitis such as *Porphyromonas gingivalis*, *Treponema denticola*, and *Tannerella forsythia* is necessary to validate its antimicrobial effect [9].

In periodontal disease, persistent and chronic inflammation leads to tissue destruction. The excessive production of cytokines and eicosanoids by infiltrated immune cells accelerates the secretion of proteolytic enzymes and promotes bone resorption. In vitro studies have shown that IL-1β and TNF-α upregulate MMPs expression in gingival fibroblasts [10], while IL-1β, TNF-α, and PGE_2_ increase osteoclast formation [47,48]. Therefore, pharmacological intervention to inhibit the production of inflammatory mediators is essential to prevent periodontal tissue destruction. In the present study, we assessed the levels of inflammatory mediators including TNF-α, IL-1β, and PGE_2_ in gingival tissue. The elevated levels of TNF-α, IL-1β, and PGE_2_ observed in the EPD control group were significantly reduced by LRC administration (Figure 6). Moreover, histopathological analyses revealed that the increased numbers of infiltrated immune cells from ligation placement were diminished in the LRC administration groups (Table 2). In LPS-stimulated RAW 264.7 cells, LRC treatment significantly reduced the production of inflammatory mediators including PGE_2_, IL-1β, TNF-α, and MCP-1 (Figure 1). Furthermore, our results demonstrated that the anti-inflammatory effects of LRC were mediated by the inhibition of MAPK and NF-κB signaling pathways (Figure 2). These findings suggest that LRC has a potent anti-inflammatory role in periodontitis by inhibiting the expression of inflammatory mediators.

As discussed above, the loss of connective tissue and alveolar bone is a significant hall mark of periodontitis progression. During the inflammatory process, the degradation of periodontal extracellular matrix is mediated by proteolytic enzymes such as MMPs, elastase, and tryptase from host cells. In particular, MMP-8 has been reported to have elevated expression levels in patients with periodontal disease and has also been found to be associated with pocket depth [11,49]. In the present study, we found that the increased gingival level of MMP-8 by ligation placement was significantly reduced by LRC administration (Figure 8). Furthermore, the decreased collagen-occupied area observed in the EPD group was significantly restored by LRC administration (Table 2).

Alveolar bone destruction is mediated by osteoclasts, the principal bone resorptive cell. The binding of RANKL on osteoblasts to RANK, a receptor expressed in osteoclast precursor cells, induces osteoclast differentiation and activation, leading to bone loss. This interaction between RANKL and RANK can be abrogated by OPG, a decoy receptor of RANKL. In an experimental ligature-induced periodontitis model, subcutaneous injection of OPG blocked the alveolar bone loss [50]. As a key regulator of osteoclastogenesis and bone resorption, the RANKL/OPG ratio is considered as a biomarker of periodontitis [39]. In our experiment, the increase in RANKL/OPG mRNA expression ratio by ligature placement in the EPD group was significantly reduced by LRC administration (Figure 9). Moreover, the increased numbers and occupied regions of osteoclasts on the alveolar bone surface by ligation placement were also significantly reduced by LRC administration (Table 3). Accordingly, the recovery of alveolar bone loss scores by LRC administration was also observed (Figure 4). Consistent with these findings, LRC inhibited RANKL-induced osteoclastogenesis in RAW 264.7 cells in a previous report [25]. Taken together, these findings suggest that LRC has potential preventive effects against bone loss associated with osteoporosis and periodontitis by inhibiting osteoclastogenesis and regulating RANKL/OPG expression. Further studies that directly evaluate alveolar bone loss and recovery using radiographic imaging will enable a more accurate assessment of LRC’s effects on periodontal destruction.

During periodontal pathogenesis, exposure to proinflammatory cytokines such as IL-1, IL-6, and TNF-α stimulates RANKL expression in osteoblasts [51]. In a rat ligature model, the exogenous application of cytokines including IL-1β and TNF-α not only intensified inflammation, but also accelerated alveolar bone destruction [52,53]. Moreover, exogenously applied PGE_2_ increased alveolar bone resorption by increasing osteoclasts in the periodontal tissue of rats [54]. Conversely, the local injection of chemical inhibitors targeting IL-1, TNF-α, and PGE_2_ decreased the resorption of alveolar bone in chronic periodontal disease models [55,56]. Therefore, these findings indicate that the inhibitory effects of LRC on the expression of inflammatory mediators can contribute to the prevention of alveolar bone loss.

Recently, oxidative stress has been increasingly recognized as a key factor in the pathogenesis of periodontitis. After initiation of the host response against pathogenic bacteria, PMNs are the most abundant inflammatory cells in the periodontal pocket and gingival cervices serving as a primary source of ROS [14]. ROS, generated by the response of neutrophils to periodontal pathogens, can be effective in killing microbes but also induce cytotoxicity to host cells through lipid peroxidation, protein damage, and DNA damage [14]. Studies have demonstrated that the peripheral neutrophils from periodontitis patients produce more ROS compared to those from healthy individuals [57]. This suggests that hyperactive neutrophils in periodontitis enhance oxidative burst and ROS release, exacerbating tissue inflammation and destruction. Consequently, levels of MDA, a well-established byproduct of lipid peroxidation, were found to be significantly higher in the gingival crevicular fluid, saliva, and serum of periodontitis patients compared to healthy individuals [14,58]. Therefore, studies validating the potential of MDA levels as a clinical biomarker of oxidative stress in periodontitis patients have been conducted [58,59]. In the present study, we showed that the activity of MPO, an enzyme produced by PMNs, was elevated in gingival tissue by ligation placement as previously reported [29,60]. The influx of PMNs in gingival tissues can be determined by assessing MPO activity. We found that LRC administration significantly reduced the elevated MPO activity observed in the EPD group (Figure 5b). Additionally, our results showed that LRC significantly inhibited the ligation-induced increase in MDA levels in gingival tissues (Figure 7b). Similarly, it has been reported that LRC application exerted an MDA-lowering effect against lipid peroxidation in mice skin [61]. Moreover, the antioxidant properties of LRC have been demonstrated in various experimental disease models including ethanol-induced gastric ulcer mice and dopaminergic neuronal cell death [24,62].

Kukoamines, phenolic alkaloids, are recognized as the predominant constituents of LRC and are used as markers for quality assessment of LRC [63]. Therefore, to ensure the quality of raw materials, we confirmed the kukoamine B content in the LRC extract using HPLC analysis (Supplementary Figure S1) before conducting efficacy tests. Additionally, as key bioactive constituents of LRC, kukoamines have been studied for their biological activities. Notably, kukoamines have been highlighted for their anti-inflammatory properties in various experimental models. For example, kukoamine A has demonstrated an anti-inflammatory effect in LPS-stimulated RAW 264.7 cells, osteoarthritis mice, and radiation-induced neuroinflammation in rats [64,65,66]. Kukoamine B has also shown an anti-inflammatory effect in LPS-induced septic mice and high fat and fructose fed mice [67,68]. In particular, kukoamine B has been considered as a potential candidate for treating sepsis and approved for clinical trials in China [69]. It neutralized LPS and CpG, which are major pathogenic molecules causing sepsis, by binding to them with high affinity, thereby preventing their interaction with toll-like receptors on macrophages [70,71]. In addition to anti-inflammatory effects, trans-*N*-caffeoyltyramine and kukoamine have shown protective effects against hydrogen peroxide-induced oxidative stress [72,73]. Moreover, kukoamine A and B exerted antioxidant and cytoprotective effects in Fenton-induced damage through radical scavenging effects [74]. Furthermore, scopolin and kukoamine A and B have shown the regulatory effects on the osteoblast or osteoclast differentiation in ovariectomized mice models [75,76,77]. Therefore, this evidence suggests that LRC can effectively improve periodontitis based on the biological activities of its bioactive constituents, particularly kukoamines.

## 5. Conclusions

In summary, our investigation revealed that LRC effectively inhibited ligation-induced anaerobic bacteria proliferation, immune cell infiltration, production of inflammatory mediators, and oxidative stress. Moreover, LRC administration ameliorated the degradation of connective tissue and the loss of alveolar bone associated with periodontitis. These findings suggest that LRC is a promising medicinal herb for alleviating periodontitis and related alveolar bone loss through its antimicrobial, anti-inflammatory, and antioxidant properties, and it could potentially be developed as a functional food for preventive measure in dental health.

## Figures and Tables

**Table 1:** Information of oligonucleotides used in RT-qPCR.

Target Gene	Orientation	Sequence (5′–3′)	NCBI Accession No.
*iNOS*	Sense Antisense	GACAAGCTGCATGTGACATC, GCTGGTAGGTTCCTGTTGTT	NM_001313922.1
*COX-2*	Sense Antisense	TCCAGATCACATTTGATTGA, TCTTTGACTGTGGGAGGATA	NM_011198.5
*TNF-α*	Sense Antisense	ATGAGCACAGAAAGCATGAT, TACAGGCTTGTCACTCGAAT	NM_013693.3
*IL-1β*	Sense Antisense	ATGGCAACTGTTCCTGAACT, CAGGACAGGTATAGATTCTT	NM_008361.4
*MCP-1*	Sense Antisense	TGATCCCAATGAGTAGGCTGG, ATGTCTGGACCCATTCCTTCT	NM_011333.3
*GAPDH*	Sense Antisense	AACGACCCCTTCATTGAC, TCCACGACATACTCAGCAC	NM_001411843.1
*RANKL*	Sense Antisense	CTGATGAAAGGAGGGAGCAC, GAAGGGTTGGACACCTGAATGC	NM_057149.2
*OPG*	Sense Antisense	TCCTGGCACCTACCTAAAACAGCA, ACACTGGGCTGCAATACACA	U94331.1
*β-actin*	Sense Antisense	TCAGGTCATCACTATCGCCAAT, AAAGAAAGGGTGTAAAACGCA	NM_031144.3

RT-qPCR, reverse transcription quantitative polymerase chain reaction; *iNOS*, *inducible nitric oxide synthase*; *COX-2*, *cyclooxygenase-2*; *TNF-α*, *tumor necrosis factor-α*; *IL-1β*, *interleukin-1β*; *MCP-1*, *monocyte chemoattractant protein-1*; *GAPDH*, *glyceraldehyde-3-phosphate dehydrogenase*; *RANKL*, *receptor activator of nuclear factor-κB ligand*; *OPG*, *osteoprotegerin*.

**Table 2:** Histological scores and histomorphometrical analysis of maxillary regions around ligation placement—gingival tissues.

Group	In Gingival Tissues		
	Histological Scores (Max =3)	Inflammatory Cells (cells/mm^2^)	Collagen Fibers (%/mm^2^)
Intact vehicle	0.30 ± 0.48	58.60 ± 17.02	76.50 ± 10.58
EPD	2.90 ± 0.32 **	743.40 ± 67.48 **	14.16 ± 4.20 **
IND (5 mg/kg)	1.50 ± 0.53 ^##^	257.40 ± 94.73 ^##^	51.20 ± 10.11 ^##^
INP (63 mg/kg)	2.10 ± 0.57 ^#^	532.80 ± 100.41 ^##^	31.48 ± 7.46 ^##^
LRC (50 mg/kg)	2.00 ± 0.67 ^##^	524.90 ± 101.41 ^##^	31.54 ± 10.01 ^##^
LRC (100 mg/kg)	1.70 ± 0.48 ^##^	387.80 ± 110.89 ^##^	41.86 ± 12.35 ^##^
LRC (200 mg/kg)	1.40 ± 0.52 ^##^	242.20 ± 84.66 ^##^	52.16 ± 10.29 ^##^

Values are expressed as mean ± SD of 10 rats. Significant versus intact vehicle control group, ** *p* < 0.01; versus EPD control group, ^#^ *p* < 0.05, ^##^ *p* < 0.01.

**Table 3:** Histomorphometrical analysis of maxillary regions around ligation placement—alveolar bone areas.

Group	In Alveolar Bone Regions		
	Alveolar Bone Volume (%)	Osteoclast Cell (cells/mm^2^)	OC/BS (%)
Intact vehicle	78.59 ± 6.50	7.80 ± 2.57	3.89 ± 1.47
EPD	20.65 ± 6.22 **	51.40 ± 7.72 **	68.42 ± 6.45 **
IND (5 mg/kg)	53.30 ± 6.41 ^##^	26.00 ± 5.89 ^##^	29.20 ± 8.95 ^##^
INP (63 mg/kg)	36.80 ± 4.61 ^##^	37.40 ± 2.84 ^##^	47.56 ± 6.20 ^##^
LRC (50 mg/kg)	36.76 ± 7.69 ^##^	37.20 ± 3.55 ^##^	47.45 ± 7.31 ^##^
LRC (100 mg/kg)	41.78 ± 6.92 ^##^	31.60 ± 5.48 ^##^	36.05 ± 6.83 ^##^
LRC (200 mg/kg)	53.89 ± 12.74 ^##^	25.80 ± 3.82 ^##^	29.10 ± 11.92 ^##^

Values are expressed as mean ± SD of 10 rats. OC/BS, the percentage of osteoclast cell-occupied regions on the alveolar bone surface. Significant versus intact vehicle control group, ** *p* < 0.01; versus EPD control group, ^##^ *p* < 0.01.

## Data Availability

The raw data supporting the conclusions of this article will be made available by the authors on request.

## Supplementary Materials

The following supporting information can be downloaded at https://www.mdpi.com/article/10.3390/antiox13111332/s1, Figure S1: Identification of kukoamine B in LRC using high performance liquid chromatography (HPLC) analysis; Figure S2: Effects of LRC on the viability of HaCaT, HDFn, and RAW 264.7 cells.
