# Supplementary material for: The Effect of the Root Bark of Lycium chinense (Lycii Radicis Cortex) on Experimental Periodontitis and Alveolar Bone Loss in Sprague-Dawley Rats
Source: Antioxidants (Basel). 2024 Oct 31;13(11):1332. doi: 10.3390/antiox13111332 (PMC11590933; doi:10.3390/antiox13111332)
Supplement: Supplementary file 1 [file antioxidants-13-01332-s001.zip › antioxidants-3268370-supplementary.pdf]

antioxidants

Supplementary Information

The Effect of the Root Bark of *Lycium chinense* (Lycii Radicis Cortex) on Experimental Periodontitis and Alveolar Bone Loss in Sprague-Dawley Rats

Jinwon Yang [1,†], Hyosun Song [2,†], Jeongjun Lee [3], Hunsuk Chung [3], Young-Sam Kwon [2], Kyung-Hwan Jegal [4], Jae-Kwang Kim [5,*] and Sae-Kwang Ku [1,*]

[1] Department of Anatomy and Histology, College of Korean Medicine, Daegu Haany University, Gyeongsan 38610, Republic of Korea; yangjinwon@dhu.ac.kr
[2] Department of Veterinary Surgery, College of Veterinary Medicine, Kyungpook National University, Daegu 41566, Republic of Korea; legendx0070@knu.ac.kr (H.S.); kwon@knu.ac.kr (Y.-S.K.)
[3] GAPI BIO Co., Ltd., Hwaseong 18622, Republic of Korea; orglab@gapibio.co.kr (J.L.); hunsukchung@dongbangchem.co.kr (H.C.)
[4] Department of Korean Medical Classics, College of Korean Medicine, Daegu Haany University, Gyeongsan 38610, Republic of Korea; jegalkh@dhu.ac.kr
[5] Department of Physiology, College of Korean Medicine, Daegu Haany University, Gyeongsan 38610, Republic of Korea
* Correspondence: kim-jk@dhu.ac.kr (J.-K.K.); gucci200@dhu.ac.kr (S.-K.K.)
† These authors contributed equally to this work.

1. Manufacturing process of Lycii Radicis Cortex extract (LRC)

The extraction process for LRC begins with a primary extraction using a 30% ethanol solution at a 10:1 solvent to sample ratio, conducted at 85°C for 8 h, This is followed by a secondary extraction with the same 30% ethanol solution but at a 7:1 solvent to sample ratio, also at 85°C for 4 h. The resulting solution is then microfiltered. Next, the filtrate is concentrated at 60–65°C until it reaches at a Brix level of 15–20. Finally, the concentrated extract undergoes spray drying with an inlet temperature of 175°C and an outlet temperature of 80°C.

2. Identification of kukoamine B in LRC using high-performance liquid chromatographic (HPLC) analysis

For standardization of LRC raw material, kukoamine B was identified using HPLC analysis. Accurately weighed 35 mg of kukoamine B, purchased from BioFron Inc. (La Mirada, CA, USA), was dissolved in 40% methanol (with 0.5% acetic acid) to prepare a 50 mL standard solution. To prepare the test samples, 100 mg of LRC powder was dissolved in 15 mL of 40% methanol (with 0.5% acetic acid). The solution was then placed in an ultrasonic shaker until fully extracted, and additional 40% methanol (with 0.5% acetic acid) was added to make the total volume 25 mL. Finally, sample solution was filtered using 0.45 μm membrane filter. 10 μL of standard and sample solution were analyzed using Agilent HPLC system (1260 Infinity II, Waldbronn, Germany) equipped with C18 column (ZORBAX Eclipse Plus C_{18}, 4.6 × 250 mm, 5 μm) at 40°C column temperature. The mobile phase was consisted of 0.05% TFA (A) and acetonitrile (B). HPLC gradient conditions were follows: 0 min (A 93%, B 7%), 5 min (A 93%, B 7%), 40 min (A 87%, B 13%), 45 min (A 0%, B 100%), 55 min (A 0%, B 100%) with the 1.0 mL/min flow rate. Absorbance at wavelength of 280 nm was detected. In the result of HPLC analysis, one peak of LRC sample solution matched with kukoamine B standard at retention time of 24.8 min (Fig. S1). Quantification of peak area indicated that LRC contains 23 mg/g of kukoamine B.

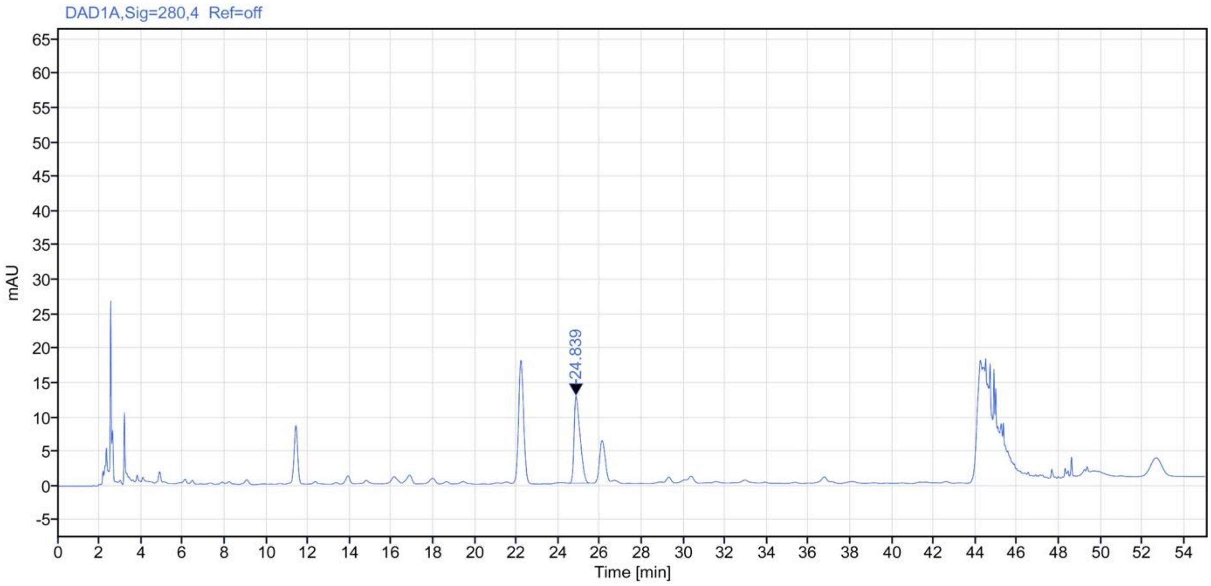

Figure S1. Identification of kukoamine B in LRC using high performance liquid chromatography (HPLC) analysis.

3. Cytotoxicity of LRC

Prior to evaluating the anti-inflammatory effect of LRC in lipopolysaccharide (LPS)-stimulated RAW 264.7 cells, cytotoxicity of LRC on HaCaT, HDFn, and RAW cells were assessed. HaCaT (a human keratinocyte cell), HDFn (a human primary dermal fibroblast-neonatal cell), and RAW 264.7 (a murine macrophage-derived cell) cells were obtained from American Type Culture Collection (ATCC; Rockville, MD, USA). HaCaT and RAW 264.7 cells were maintained in Dulbecco's modified Eagle's medium (DMEM; HyClone Laboratories, Logan, UT, USA) with 10% fetal bovine serum (Lonza, Walkersville, MD, USA) and antibiotics. HDFn cells were cultured in fibroblast basal medium (FBM; ATCC), supplemented with FBM low serum kit (ATCC). Cells were cultured at 37°C in a humidified atmosphere with 5% CO_2. For cell viability assay, three types of cells were plated at a density of 5×10^3 cells per well in a 48-well plate. After serum starvation for 3 h, cells were treated with LRC from 0.001 to 10 mg/mL for 24 h. The viable cells were stained with 3-(4,5-Dimethylthiazol-2-yl)-2,5-diphenyltetrazolium bromide (MTT; Sigma-Aldrich) solution (0.1 μg/mL for 4 h). After the media was removed, formed formazan crystals in the wells was dissolved by adding dimethyl sulfoxide. Absorbance was measured at 570 nm using an automatic microplate reader (EnSpire™; PerkinElmer, Waltham, MA, USA). Relative cell viability was determined by dividing the absorbance of the treated sample by the absorbance of the control and then multiplying the result by 100. As a results, no significant changes on the survivability of HaCaT, HDFn, and RAW 264.7 cells by LRC treatment have observed.

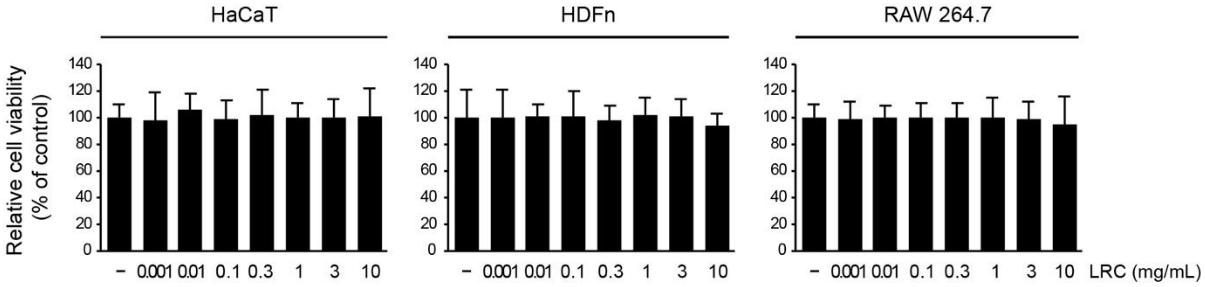

Figure S2. Effects of LRC on the viability of HaCaT, HDFn, and RAW 264.7 cells. HaCaT (human keratinocyte cell, left), HDFn (human primary dermal fibroblast-neonatal cell, middle), and RAW 264.7 (a murine macrophage-derived cell) cells were treated with LRC from 0.001 to 10 mg/mL for 24 h. Relative cell viability was measured by MTT assay.
